# Microbiome changes in the sponge *Halichondria panicea* along the Baltic Sea salinity gradient

**DOI:** 10.3389/fmicb.2025.1723082

**Published:** 2026-01-27

**Authors:** Philipp Hoy, Christiane Hassenrück, Felix Mittermayer-Schmittmann, Lara Schmittmann, Klaus Jürgens

**Affiliations:** 1 Leibnitz Institute for Baltic Sea Research Warnemünde (IOW), Rostock, Germany; 2 GEOMAR Helmholtz Centre for Ocean Research Kiel, Kiel, Germany

**Keywords:** 16S rRNA metabarcoding, bacterial symbionts, Baltic Sea, demosponge, *Halichondria panicea*, marine sponge, microbiome, salinity gradient

## Abstract

The Baltic Sea is characterized by a strong salinity gradient, which impacts the diversity and composition of free-living macro- and microorganisms. Much less is known about how host-associated microorganisms are affected by decreasing salinities. Marine sponges are known to harbor complex prokaryotic communities, relevant for the host’s health and functions. This raises the question of whether and how the sponge microbiomes are also subject to changes along the declining salinity of the South-western Baltic Sea. We analysed the microbiome of the dominating sponge, the marine demosponge *Halichondria panicea,* from high saline conditions (28.3 PSU) in the Kattegat to the Eastern limit of its distribution at lower salinities (11.4 PSU). We utilized a dual approach of 16S rRNA gene metabarcoding of sponge and seawater microbiomes, together with the absolute quantification of the total prokaryotic sponge community and the main bacterial symbiont, the alpha-proteobacterium *Ca. Halichondribacter symbioticus* (*Ca.* H.s.), by digital droplet PCR (ddPCR). All sponge microbiomes originating from the same location shared a highly similar prokaryotic community, clearly different from the surrounding water, and dominated by the known symbiont *Ca.* H.s. In addition, location-specific bacterial genera, enriched in the sponge microbiomes, were also found (e.g., *Endozoicomonas, Shewanella, Ekhidna*). Sponge microbiomes at the Eastern limit of the distribution of *H. panicea*, with the lowest salinity, showed a higher sponge microbiome diversity and an increased similarity between sponge and water microbiomes. Furthermore, at the lowest salinity, absolute bacterial abundance increased while *Ca.* H.s. remained at relatively stable absolute abundances. These observations were paired with a notable trend of decreasing body volume of sponge individuals, indicating lower fitness at the lowest salinities. We hypothesize that the observed shifts under low salinity conditions, with an increased occurrence of bacterial taxa from the surrounding water, reflect potential early signs of dysbiosis of the sponge microbiome, coinciding with the occurrence at their distribution limit. This study provides a first insight into the effects of changing salinity on the microbiome of *H. panicea* in the Baltic Sea, and on the shifts in sponge microbiomes that occur in environmental gradients such as the challenging environment of the Baltic Sea.

## Introduction

The Baltic Sea is one of the largest brackish water bodies in the world. Two antagonists create the East–West and North–South gradient: Inflow events via the Danish straits of saline seawater and the constant freshwater runoff from over 200 rivers. This spatial salinity gradient, extending about 2000 km from the Kattegat to the Bothnian Bay, forms a highly variable and physiologically demanding environment for its inhabitants and impacts their geographic distribution (Snoeijs-Leijonmalm and Andrén, 2017). For example, as already proposed by Remane (1934), the alpha diversity of marine macrozoobenthos strongly declines with decreasing salinity (Zettler et al., 2014). However, the trend of declining alpha diversity does not necessarily translate to all organisms inhabiting the Baltic Sea. For example, richness and diversity indices of the bacterioplankton do not show consistent shifts along the entire salinity gradient of the Baltic Sea despite the occurrence of strong, salinity-induced changes in the community composition (Herlemann et al., 2011). Whereas free-living microorganisms in the Baltic Sea have been examined in many studies, much less is known for microbial taxa that live in close relationship with and within multicellular organisms (Bosch and McFall-Ngai, 2011). Such “holobionts,” consisting of a host organism and associated symbionts, are important biological units in marine ecosystems where they perform functions in nutrient or carbon cycling that cannot be accomplished by the partners separately (Pita et al., 2018; González-Pech et al., 2024).

One group of organisms, for which associated microorganisms are particularly relevant, is marine sponges. Sponges are important components of marine benthic communities, contributing to many ecosystem functions such as habitat building and, due to their high filtration capacity, nutrient cycling and bentho-pelagic coupling (Bell et al., 2020). The microbiome plays an important role for their sponge hosts, including chemical defence, host nutrition, and removal of contaminants or metabolic waste (Webster and Thomas, 2016; Pita et al., 2018). Most sponge species can host a highly diverse and abundant community of microorganisms (bacteria, archaea, fungi, viruses, and microalgae), sometimes exceeding several billion microbial cells per gram of wet sponge weight (Gloeckner et al., 2014). Sponge microbiomes are often highly specific to certain sponge species (Hentschel et al., 2012; Thomas et al., 2016; Busch et al., 2022a), with some microorganisms exclusively occurring in sponges or being highly enriched compared to the surrounding seawater (Taylor et al., 2013). In terms of community structure, complex host-associated microbial communities could be divided into a core microbiome (taxa that are prevalent in all individuals of the same host species) and a variable, transient microbiome (microbial taxa that are found only in some individuals or that vary in their relative abundance) (Schmitt et al., 2012; Thomas et al., 2016).

Changes in environmental factors may alter the relationships between sponges and their symbionts, which could potentially influence the sponge abundance and distribution patterns (Busch et al., 2022a). There is also evidence indicating that environmental stress, such as increased temperature or pollution impact, sponge-associated microbial diversity, particularly the variable fraction (Pita et al., 2018). Disturbances can disrupt the taxonomic composition of the microbiome, inducing a microbial dysbiosis. This can involve a shift from sponge-specific taxa to opportunists, with implications for the health and physiological functions of the host (Pita et al., 2018; Vad et al., 2022; Botté et al., 2023). Similar alterations in sponge microbiomes may occur at the edge of the species distribution where environmental factors become unfavorable. However, we are lacking sufficient data on natural environmental gradients that can serve as a baseline to monitor future changes in sponge holobionts.

The sponge *Halichondria panicea* (breadcrumb sponge) inhabits coastal areas worldwide, including parts of the brackish Baltic Sea, and is therefore a great model to study microbiome changes along a natural environmental gradient. *Halichondria panicea* is a so-called low microbial abundance (LMA) sponge that, in contrast to high microbial abundance (HMA) sponge species, is characterized by a lower microbial diversity and abundance. LMA sponges are often dominated by a few bacterial taxa specific to their hosts, as is the case for the *H. panicea* microbiome that is dominated by an alphaproteobacterium “*Candidatus* Halichondribacter symbioticus” (hereafter *Ca.* H.s.) (Knobloch et al., 2019). This symbiont was confirmed to be associated with *H. panicea* at various locations across the North Atlantic (Althoff et al., 1998; Naim et al., 2014; Steinert et al., 2017; Wichels et al., 2006), and also the Baltic Sea (Schmittmann et al., 2022), often constituting more than half of the bacterial abundance in the sponge body. Genome analysis of *Ca.* H.s. revealed putative beneficial traits for the sponge host, such as synthesis of vitamin B12 and bacteriocins, taurine, and sulfoacetate metabolism (Knobloch et al., 2020).

*Halichondria panicea* is one of the most abundant sponge species found in the western Baltic Sea (Barthel, 1991), extending in easterly distribution approximately until the island of Rügen (Zettler et al., 2018). Previous microbiome analyses of *H. panicea* from the Baltic Sea are limited to samples from Kiel Bight (Althoff et al., 1998; Schmittmann et al., 2022). We do not have information, however, on whether and how the microbiome of *H. panicea* is changing along the salinity gradient of the Baltic Sea. Given the fact that the composition of bacterioplankton changes strongly along the salinity gradient of the Baltic Sea (Herlemann et al., 2011), it is reasonable to presume that sponge microbiomes must also be influenced by the decreasing salinity. Comprehensive knowledge of how the sponge holobiont is influenced by natural environmental stressors is necessary in order to better understand the effects of the diverse anthropogenic stressors, which are prevalent in the Baltic Sea (Reusch et al., 2018), on the sponge holobiont.

The objective of this study was therefore a first assessment of the *H. panicea* microbiome along the unique salinity gradient of the Baltic Sea. Particularly, we aimed to study:

(1) whether and how the relative composition and diversity of the microbiome is changing at different salinities
(2) whether the abundance and contribution of the dominant symbiont *Ca.* H.s. to the microbiome is changing along the salinity gradient
(3) whether sponge-specific prokaryotes other than *Ca.* H.s. can be identified in the microbiome of *H. panicea*

To achieve these goals, we used a combined approach of 16S rRNA gene amplicon sequencing of sponge and seawater microbiomes, along with the quantification of 16S rRNA gene copies of total bacteria and the dominant symbiont in the sponge microbiome by ddPCR (digital droplet PCR). We analysed the variability between sponge individuals originating from the same location, the overlap between the prokaryotic seawater community and the sponge microbiome, as well as the abundance of the main sponge symbiont, *Ca.* H.s.

## Materials and methods

### Sampling locations and collection

**Figure 1:**
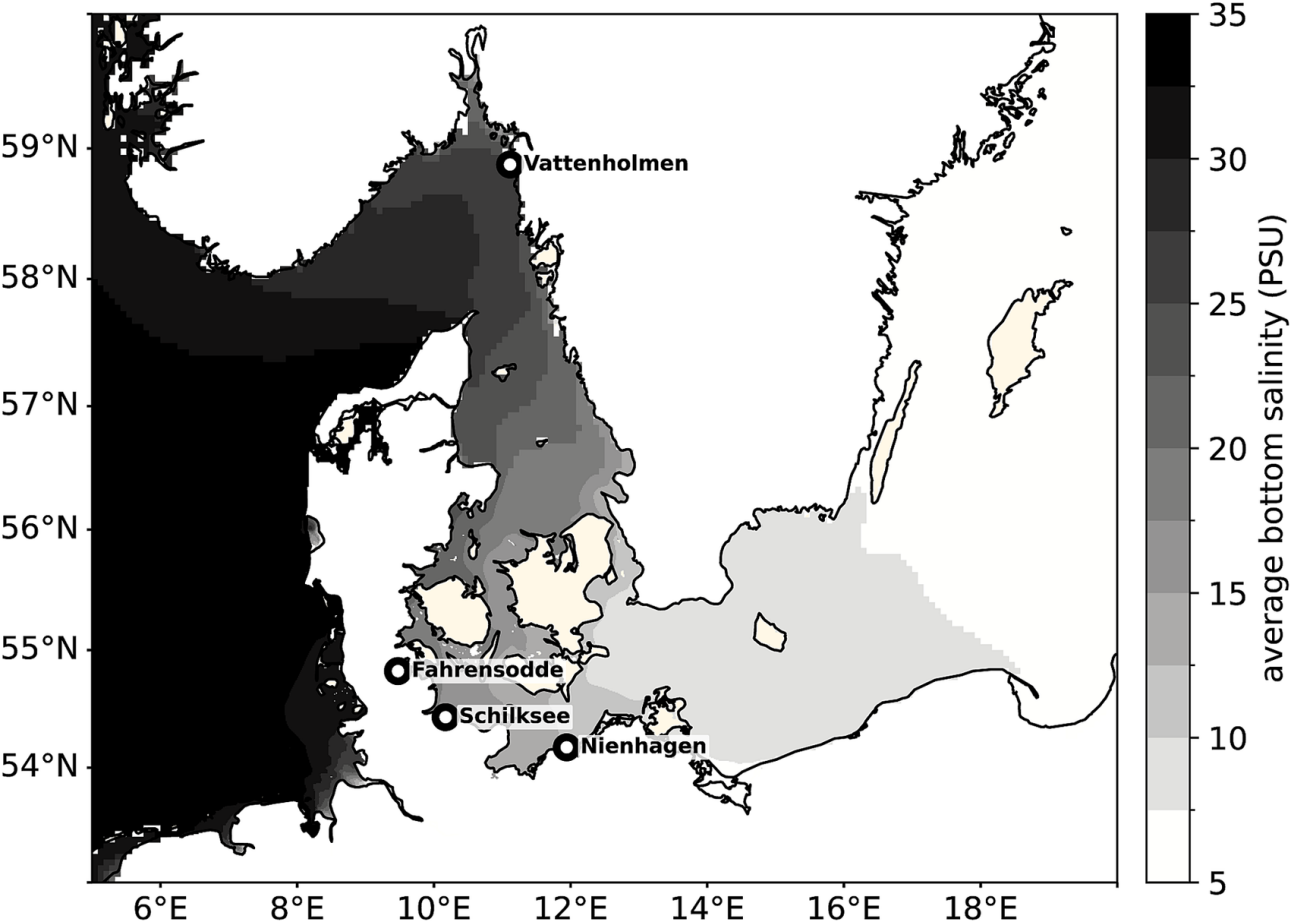
Sampling locations with average bottom salinity in the Western Baltic Sea. Average bottom salinity at the sampling stations was derived from simulated salinity fields provided by the BSH-HBMnoku circulation model for the North and Baltic Seas (Brüning et al., 2021). Twelve-hourly salinity values were extracted from the deepest valid model grid cells and averaged over a 9-year period (2016–2024).

Sponge tissue and seawater samples were collected from four locations along the Swedish and German coastline of the Baltic Sea and Kattegat by snorkelling and scuba diving (Figure 1). Sampling at the Swedish location (Vattenholmen) took place in May 2018, while sampling for the three other locations in German waters was performed in April 2023. *Halichondria panicea* was identified based on known morphological characteristics (Ackers et al., 2007), and sponges were picked several meters apart from one another to ensure each sample originated from a different individual. Clean sponges with minimal contamination, such as ingrown mussels and algae, and with a bright yellow coloring were selected. Pieces of approximately 10–15 cm^3^ sponge tissue were sampled from 10 individuals per station with a spatula and transferred directly into a sampling tube. For examining the water microbiomes, three seawater samples were taken per station at approximately the same water depth as the corresponding sponge colonies in plastic fish bags. After underwater sample collection, the sponge tissue and seawater samples were stored on ice for a maximum of 5 h until further processing.

### Sample processing

Sponge tissue samples taken during the first sampling campaign at Vattenholmen, Sweden, were rinsed with sterile filtered seawater in a petri dish. Algae and other contaminations were carefully removed with sterilized forceps and scalpel, and the sponge tissue was drained on an autoclaved paper towel before being fragmented and fixed in RNAlater solution (Invitrogen). A volume of 2 L of each seawater sample was filtered (0.22 μm pore-size PVDF, Merck Millipore, Germany) for DNA extraction and fixed in RNAlater solution. Sponge tissue and filters were stored for 24 h at 4 °C to complete fixation, before being transferred to −80 °C for long-term storage.

Sponge tissue samples from the other locations, taken during the second sampling campaign in German waters, were largely processed according to the protocol of Busch et al. (2022b). The samples were also cleaned of contamination using forceps and a scalpel. The cleaned sponge tissue was rinsed with sterile seawater by shaking in a 50 mL conical tube for approximately 20 s. The rinsed and already slightly fragmented tissue samples were drained on an autoclaved paper towel before the last visible contaminations were removed. Cleaned tissue samples were cut into small pieces, well mixed, subsampled, and flash frozen in liquid nitrogen before being stored at −80 °C for long-term storage. A volume of 500 mL of each seawater sample was filtered (0.22 μm pore-size white polycarbonate filter) for DNA extraction and flash frozen in liquid nitrogen.

### DNA extraction

DNA extraction from each sponge tissue sample (between 39 and 219 mg) was performed using the DNeasy PowerSoil Pro Kit (Qiagen, Germany) according to the manufacturer’s protocol. Half of each of the seawater filters was used for DNA extraction using a DNeasy Mini Kit (Qiagen, Germany), according to the manufacturer’s protocol.

### Molecular identification of sponge hosts

For each sponge tissue sample, a mitochondrial marker gene was amplified and sequenced to verify the species identification in the field. A partial sequence of the cytochrome c oxidase I gene (COI) of 628 bp length was PCR amplified using sponge-specific primers Hali LCO 3 (5′-CGAATTAATGAGTATGTATTTAAGCCGTTG-3′) and Hali HCO 4 (5′-CCAAATATTTGTTTTTTAGCAGAAAAGGTTGGTATC-3′) (Hoy, 2021). PCR products were purified using the innuPREP DOUBLEpure Kit (Analytik Jena, Germany) according to the manufacturer’s protocol. For elution of DNA, sequencing buffer of the BigDye™ Terminator v1.1 Cycle Sequencing Kit (Thermo Fisher Scientific, USA) was used. Sequencing of COI fragments was performed by the chain-termination method from both ends of the amplified fragment using the BigDye™ Terminator v1.1 Cycle Sequencing Kit in combination with a 3130xl Genetic Analyzer (Thermo Fisher Scientific, USA). Raw sequence data were automatically base-called and manually checked in the CEQ 8000 Genetic Analyser environment (Beckman & Coulter, USA). Sequences were aligned and primer-clipped in BioEdit version 7.2.5 (Hall, 1999). Each sequence variant was compared against the NCBI nucleotide database on 2024-01-03 using the BLAST function (Wheeler et al., 2007).

### 16S rRNA gene amplicon sequencing and sequence processing

DNA extracts were quantified by Qubit (DNA Broad Range Kit, Thermo Fisher Scientific, USA) and quality checked by NanoDrop (Thermo Fisher Scientific, USA). The V3–V4 hypervariable region of the 16S ribosomal RNA gene was amplified with the primers 341F (5′-CCTAYGGGRBGCASCAG-3′) and 806R (5′-GGACTACNNGGGTATCTAAT-3′) (Sundberg et al., 2013), which cover >92% of the known bacterial and archaeal diversity according to SILVA r138.2 (see Supplementary Table S1), and sequenced in a 2×300 bp paired-end run on the MiSeq Illumina platform at LGC Genomics (Berlin, Germany). Sequencing libraries were generated in mixed orientation so that both forward and reverse primers were present in both read 1 (R1) and read 2 (R2). After demultiplexing (performed by LGC Genomics), primers were removed using cutadapt version 4.2 (Martin, 2011), allowing for a mismatch rate of 0.16 and requiring an overlap of at least 16 and 19 bp for forward and reverse primers, respectively, without indels. Primer-clipped reads were then further processed in dada2 version 1.26.0 (Callahan et al., 2016) implemented in R version 4.2.2 (R Core Team, 2022), treating reads in different orientations separately. Prior to denoising, R1 and R2 reads were truncated to 260 and 200 bp, respectively, and filtered to a maximum expected error rate of 2. Error learning, denoising, and merging of denoised paired-end reads were conducted with default settings, except that the pseudo-pooling mode was selected for the denoising step. After merging, the two sequence tables generated from the different orientations were combined after reverse-complementing the amplicon sequence variants (ASVs) detected in the reverse-forward oriented part of the data set. ASVs were taxonomically classified with the R function assign Taxonomy of the dada2 package against the SILVA ribosomal reference database release 138.1 (Quast et al., 2012). ASVs shorter and longer than 380 bp and 433 bp, respectively, as well as those affiliated with eukaryotes, were removed from the data set. The ASVs, which were classified as *Amylibacter* (altogether 19 ASVs), were aligned against the NCBI nt database (accessed July 2023) to identify those most closely related to *Ca.* H.s. (MH734183.1 *Candidatus* Halichondribacter symbioticus clone Hp1 16S ribosomal RNA gene, partial sequence) from Genbank, applying a threshold of at least 99.75% sequence similarity. More details on amplicon sequence processing are available in the corresponding git repositories (Hassenrueck, 2022; Hassenrueck and Hoy, 2023).

### Absolute quantification of *Candidatus* Halichondribacter symbioticus and total bacterial numbers

To assess the absolute abundance of the total bacterial communities and of *Ca.* H.s., copy numbers of the 16S rRNA gene were measured by droplet digital PCR (ddPCR). Two primer sets originally applied to quantitative PCR (qPCR) (Schmittmann and Pita, 2022) were adapted for ddPCR (fluorescence intensity diagram: Supplementary Figure S1). E1052f (5′-TGCATGGYTGTCGTCAGCTCG-3′) and E1193r (5′-CGTCRTCCCCRCCTTCC-3′) (Wang and Qian, 2009) are universal for most bacterial taxa, while Hal Sym F (5′-CGCGGATGGTAGAGATACCG-3′) and Hal Sym R (5′-TGTCCCCAACTGAATGCTGG-3′) (Schmittmann and Pita, 2022) amplify only 16S rRNA gene fragments of *Ca.* H.s. The amplified fragments have a length of 141 and 148 bp, respectively. Measurements were performed on a QX200 Droplet Digital System (Biorad, USA) using the EvaGreen Assay according to the manufacturer’s instructions. DNA extracts for quantification of total bacterial abundance were used in a 1:1000 dilution (approx. 0.05–0.25 ng/μL), and for quantification of *Ca.* H.s. in a 1:100 dilution (approx. 0.5–2.5 ng/μL). The annealing temperature used for both primer sets was 59.4 °C. The assays were analysed in QuantaSoft™ Analysis Pro v1 by Biorad. Thresholds for minimum and maximum fluorescence signal were set manually for each sample based on the distribution of data points.

Compositional data from 16S rRNA amplicon sequencing can be used in combination with gene copy number enumeration techniques such as quantitative PCR methods to obtain estimates of copy number abundances per taxon (Zhang et al., 2022; Azarbad et al., 2022). By dividing the absolute copy numbers of the 16S rRNA gene of *Ca.* H.s. measured by ddPCR by their proportion in the 16S amplicon sequencing data, we obtained an estimate of the total number of 16S rRNA gene copies for each sample. Copy numbers of *Ca.* H.s. were chosen over copy numbers obtained with universal 16S rRNA gene primers as a reference for the abundance estimation due to better performance in the ddPCR (Supplementary Figure S1). Then, ASV proportions in each sponge sample were multiplied by the estimated total number of gene copies to yield copy number estimates per ASV, allowing for a quantitative comparison of community composition independent of the bias inherent to compositional data. To validate this approach, we compared the independently quantified 16S rRNA gene copies with the calculated estimates for the total number of gene copies, obtaining a strong correlation (Supplementary Figure S2).

### Statistical analysis

Asymptotic estimates of alpha diversity indices (richness, exponential Shannon index) were calculated using the R package “iNEXT” version 3.0.0 (Hsieh et al., 2016). Kruskal–Wallis and Dunn tests were applied to test for differences by location. *p*-values of Dunn tests were adjusted using Bonferroni correction. Beta diversity was assessed as Bray-Curtis dissimilarities based on relative 16S rRNA gene sequence proportions and calculated in R version 4.2.1 (R Core Team, 2022) using the package” vegan” version 2.6–2 (Oksanen et al., 2022). Differences in sponge microbiome composition between locations were tested by PERMANOVA (999 permutations) and pairwise ANOSIM tests. *p*-values of ANOSIM tests were adjusted using FDR correction. Differences within-location microbiome heterogeneity (beta dispersion) were assessed via the function “betadisper” of the “vegan” package and tested for statistical significance by ANOVA and a *post hoc* Tukey HSD test. Bray–Curtis dissimilarity between each sponge sample and the centroid of corresponding seawater samples was calculated using the package “usedist” version 0.4.0 (Bittinger, 2020) and tested for significant differences by location using Kruskal–Wallis and Dunn-test as post hoc test utilising the package” FSA” version 0.9.5 (Ogle et al., 2023). *p*-values of Dunn tests were adjusted using Bonferroni correction. To preclude that the observed trends were driven by the high sequence proportion of *Ca.* H.s., the analysis was repeated without *Ca.* H.s. ASVs. Kruskal–Wallis tests were applied for testing for differential enrichment of specific ASVs by location, using the calculated 16S rRNA gene copy numbers. Kruskal–Wallis tests were adjusted by FDR correction, and Dunn tests were adjusted by Holm correction. *p*-values of Dunn tests were adjusted using Bonferroni correction. All figures were generated in R (R Core Team, 2022) using base R and the package” ggplot2” (Wickham, 2016). Selected plots were optimized using the graphic design software Adobe Illustrator (USA).

## Results

### Sampling locations and sponge morphologies

The sampled locations represented a bottom salinity gradient from 28.3 at Vattenholmen (Sweden) to 11.4 PSU at Nienhagen according to measurements conducted during sampling (Table 1). This corresponded well to the modeled average bottom salinity of the past 9 years (Table 1). Water depths, temperature, substrate, and surrounding benthic communities for sampling of sponge individuals differed between the stations (Table 1). *In situ* images of sponges in their habitat and sampled sponge individuals are shown in Supplementary Figures S3, S4.

**Table 1:** Biotic and abiotic parameters of sampling locations.

Parameters	Vattenholmen	Fahrensodde	Schilksee	Nienhagen
Sampling date	2018-05-18	2023-04-04	2023-04-05	2023-04-23
Latitude	58.877944	54.823805	54.433092	54.174985
Longitude	11.114694	9.474667	10.171039	11.943486
Depth (m)	0.5–2	1–2	4–6	11–12
Salinity (PSU)	28.30	18.05	16.17	11.35
Mod. salinity (PSU)	25.10	18.69	17.38	12.46
Water Temp. (°C)	14	5	5	7
Substrate	Rock	Rock	Algae	Algae
Community	cra, hyz	bar, bm, fora, fiba	fora, fiba, hyz	fora, fiba, hyz

Cra, coralline red algae; hyz, hydrozoa; bar, barnacles; bm, blue mussel; fora, foliose red algae; fiba, filamentous brown algae.

At Vattenholmen, a small island off the Swedish coast, *H. panicea* individuals grew exposed on rocky substrate spanning loose boulders to rocky vertical drop-offs. Fahrensodde is a marina in the Flensburg Fjord. *Halichondria panicea* individuals appeared in large abundances growing in encrusting sheets on the rocky substrate of the jetty enclosing the marina. Here, at least one more sponge species was observed in close proximity to *H. panicea,* which might belong to the genus *Haliclona* based on macroscopic observations. At Kiel Fjord (Schilksee), specimens were sampled next to a marina at a wave breaker made of natural boulders. Individuals of *H. panicea* displayed more roundish and compact growth forms and were sampled when attached to foliose red algae. Suspected *Haliclona* sp. individuals were also present. In contrast to Fahrensodde, an increased amount of organic material was observed as a fluffy layer on all surfaces. In the bay of Mecklenburg, the sampling was conducted at the “Artificial reef Nienhagen” (Schygulla and Peine, 2013). Similar to the sampling location Schilksee, *H. panicea* grew mostly attached to foliose red macroalgae (from which they were sampled) and only rarely on the hard substrate that the concrete foundation of the artificial reef provides. Overall, a decrease in body size of sampled *H. panicea* individuals along the salinity gradient was observed (Supplementary Figure S4).

### COI-DNA-barcoding sponge hosts

A partial sequence of the Cytochrome c oxidase I gene (COI) of 628 bp length was generated for each of the 40 analysed sponge hosts and deposited at the NCBI nucleotide database (PP728111-PP728150). The pairwise sequence similarity for all generated sequences ranged between 99.8 and 100%. The sequences were compared against the NCBI nucleotide database, resulting in” *H. panicea* mitochondrion, complete genome (NC 040168.1)” being the best match with 99.8–100% sequence identities for all samples. These results confirmed the morphological identification of all sampled sponge individuals as *H. panicea* and revealed a low intraspecific diversity.

### Sponge and water microbiomes

Sponge and water microbiomes from all locations were generally strongly dominated (mostly >99% of total sequences) by bacteria. Maximal archaeal proportions of 1–3% were found only in sponge individuals from Vattenholmen and belonged to *Nitrosopumilaceae.* However, as a previous specific archaeal amplicon sequencing study revealed a much higher archaeal diversity associated with *H. panicea* from the Baltic Sea (von Hoyningen-Huene et al., 2024), we cannot exclude that the overall proportion and diversity of archaea were underestimated with the primers used here.

**Figure 2:**
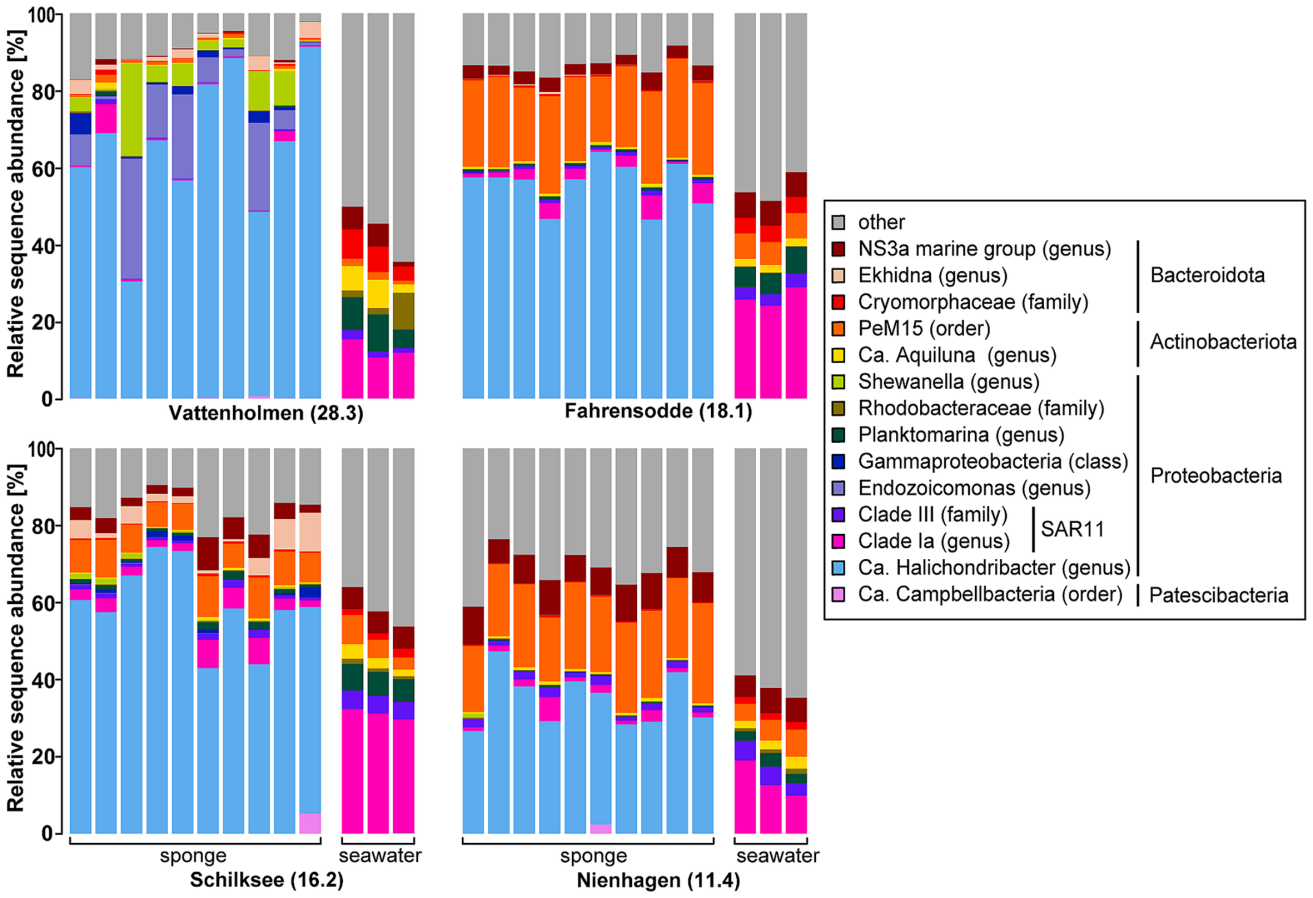
Community composition of sponge (10 individuals) and seawater (three replicates) microbiomes per location (and salinity) based on 16S rRNA gene sequence data. Taxa with a relative abundance of at least 5% at the genus level or next known higher taxonomic level (when genus was unclassified) in at least one sample are shown.

Composition of sponge microbiomes between individuals was consistent within stations and clearly different from that of the surrounding water (Figure 2). Further, they were heavily dominated by the known symbiont *Ca.* H.s. (Figure 2), comprising 26.7–91.3% of the total 16S rRNA sequences. The observed diversity in the *Ca. Halichondribacter clade* was remarkably low, with only one ASV dominating with >95% this clade and 16 additional ASVs that were >99.5% similar. Only one other, more distantly related ASV (ASV_47; 95% similarity) of the related genus *Amylibacter* was found in low relative abundance (<0.5%) in our dataset. Sponge microbiomes from Vattenholmen displayed the highest variability in the relative abundance of *Ca.* H.s. between individual sponges (30.4–91.3%, mean value: 65.9%) and differed also with respect to the other bacterial taxa from the three remaining locations. At Nienhagen, the relative abundance of *Ca.* H.s. was the lowest of all locations with a mean value of 34.5%. The mean values for Fahrensodde and Schilksee were 55.7 and 59%, respectively. In most water samples, *Ca.* H.s. was either not detected at all or comprised <0.2% of the sequences.

The dominant taxa of the seawater communities were the SAR11 Clade Ia (genus) and Clade III (family), and *Planktomarina* (all *Alphaproteobacteria*), *Ca.* Aquiluna and the order *PeM15* (*both Actinobacteria*), the *NS3a marine group*, and *Cryomorphaceae* (both *Bacteroidota*) and (*Actinobacteria*). These taxa occurred at all locations, although in varying proportions (Figure 2). At Vattenholmen, dominant seawater taxa were only found in low relative abundances (<1%) in the sponge tissues. More overlap between seawater and sponge microbiomes was observed for the three other locations. Here, dominant seawater taxa like *PeM15* (*Actinobacteriota*) and the *NS3a marine group* (*Bacteroidota*) also contributed to the sponge microbiomes. *PeM15* occurred in all seawater communities (0.1–7.6%), and the same ASVs displayed an increased relative abundance within the sponge microbiomes from Fahrensodde (17–25.7%), Schilksee (6.3–10.6%), and Nienhagen (16.5–26%) (Figure 2).

**Figure 3:**
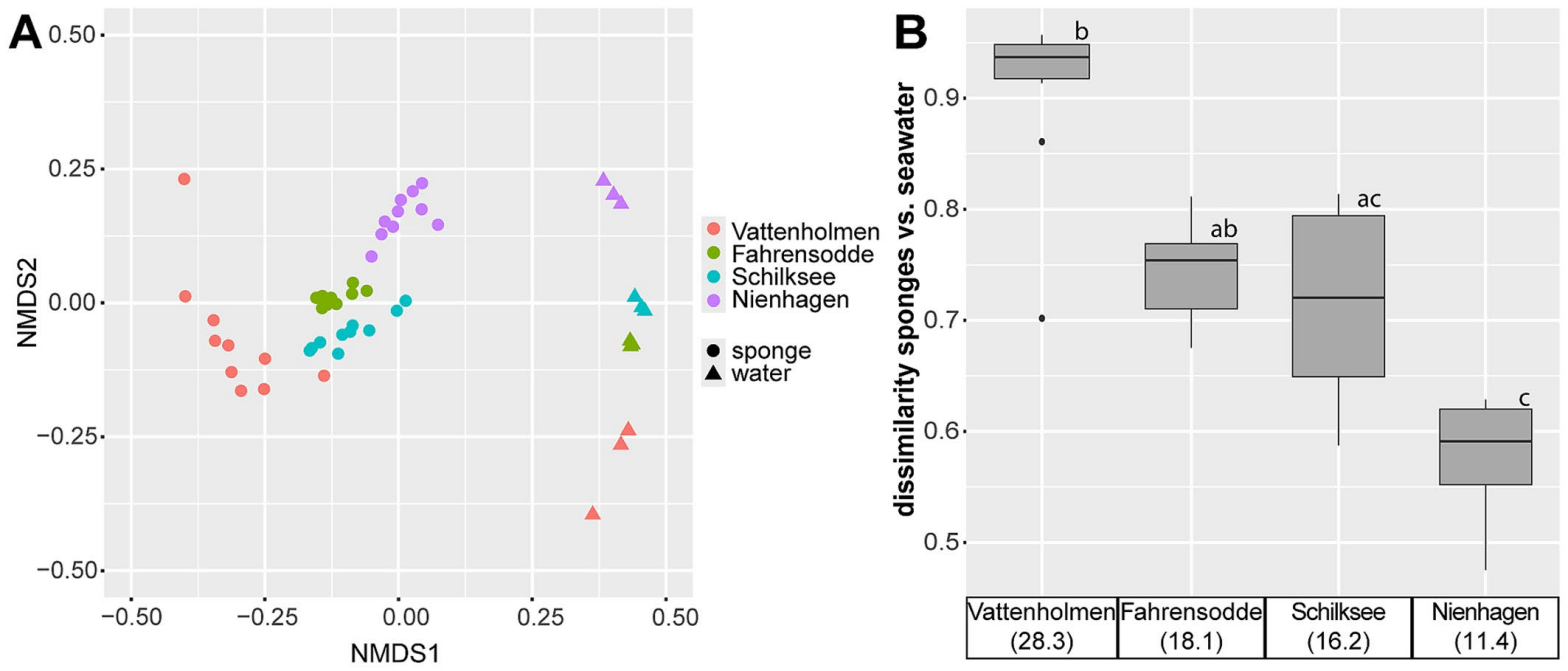
**(A)** Beta diversity of sponge and seawater microbiomes based on 16S rRNA gene sequence data. Clustering was performed using nonmetric multi-dimensional scaling (NMDS) of Bray–Curtis dissimilarities. **(B)** Bray–Curtis dissimilarities based on 16S rRNA gene sequence data between individual sponge microbiomes and centroids of corresponding seawater microbiomes per location (and salinity). Results of *post-hoc* test (Dunn) are shown in Supplementary Table S3. Different lowercase letters indicate significant differences.

Non-metric multi-dimensional scaling of Bray-Curtis dissimilarities showed a clear separation between sponge and seawater microbiomes (Figure 3A), which clustered by the corresponding locations from which they derived. Significant differences between sponge microbiomes from all locations were supported by PERMANOVA (R^2^ = 0.504, *F* = 12.197, *p* = 0.01) and pairwise ANOSIM tests (Supplementary Table S2). Furthermore, sponge microbiome heterogeneity differed significantly between locations (ANOVA: *F*_3,36_ = 11.829, *p* < 0.001). A *post-hoc* Tukey HSD test revealed a statistically significant difference between samples from Vattenholmen and all remaining locations.

Although all sponge microbiomes differed clearly from their surrounding seawater, the dissimilarity between sponge and seawater communities decreased with declining salinities (Figure 3B). A Kruskal–Wallis test revealed a significant difference in this dissimilarity between locations (*χ*^2^ = 28.092, df = 3, *p*-value < 0.001). Samples from Vattenholmen displayed the highest Bray–Curtis dissimilarity between sponge microbiomes and the seawater community, with a mean Bray–Curtis dissimilarity of 0.91. The location with the lowest dissimilarity between sponge microbiomes and seawater community was Nienhagen, with a mean Bray–Curtis dissimilarity of 0.58. Fahrensodde and Schilksee settled in between (Figure 3B). All before mentioned observations also applied when the dominant symbiont *Ca.* H.s. was excluded from the dataset (Supplementary Figure S5 and Supplementary Table S4).

**Figure 4:**
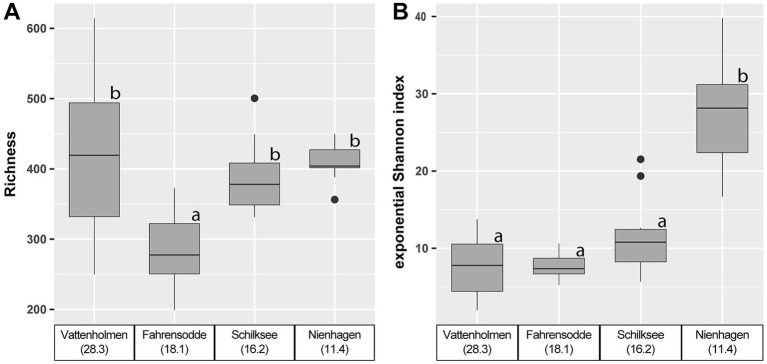
**(A)** Richness of sponge microbiomes. **(B)** Exponential Shannon index of sponge microbiomes. Both are based on 16S rRNA gene sequence data. For rarefaction curves, see Supplementary Figure S6. Different lowercase letters indicate significant differences.

Sponge microbiome alpha diversity showed significant differences by location (Kruskal–Wallis; richness: *χ*^2^ = 17.044, df = 3, p < 0.001, exponential Shannon index: *χ*^2^ = 23.039, df = 3, *p*-value = 0.001) (Figures 4A,B). However, Fahrensodde was the only location with statistically significant lower richness with a median of 277.4 ASVs (Figure 4A; Supplementary Table S5). The variance in richness between individual samples seemed to decrease from Vattenholmen to Nienhagen. The exponential Shannon index was significantly higher at Nienhagen (median 28.1) compared to the other three locations (medians: 7.4–10.8) (Figure 4B; Supplementary Table S5).

#### Quantification of *Candidatus* Halichondribacter symbioticus

**Figure 5:**
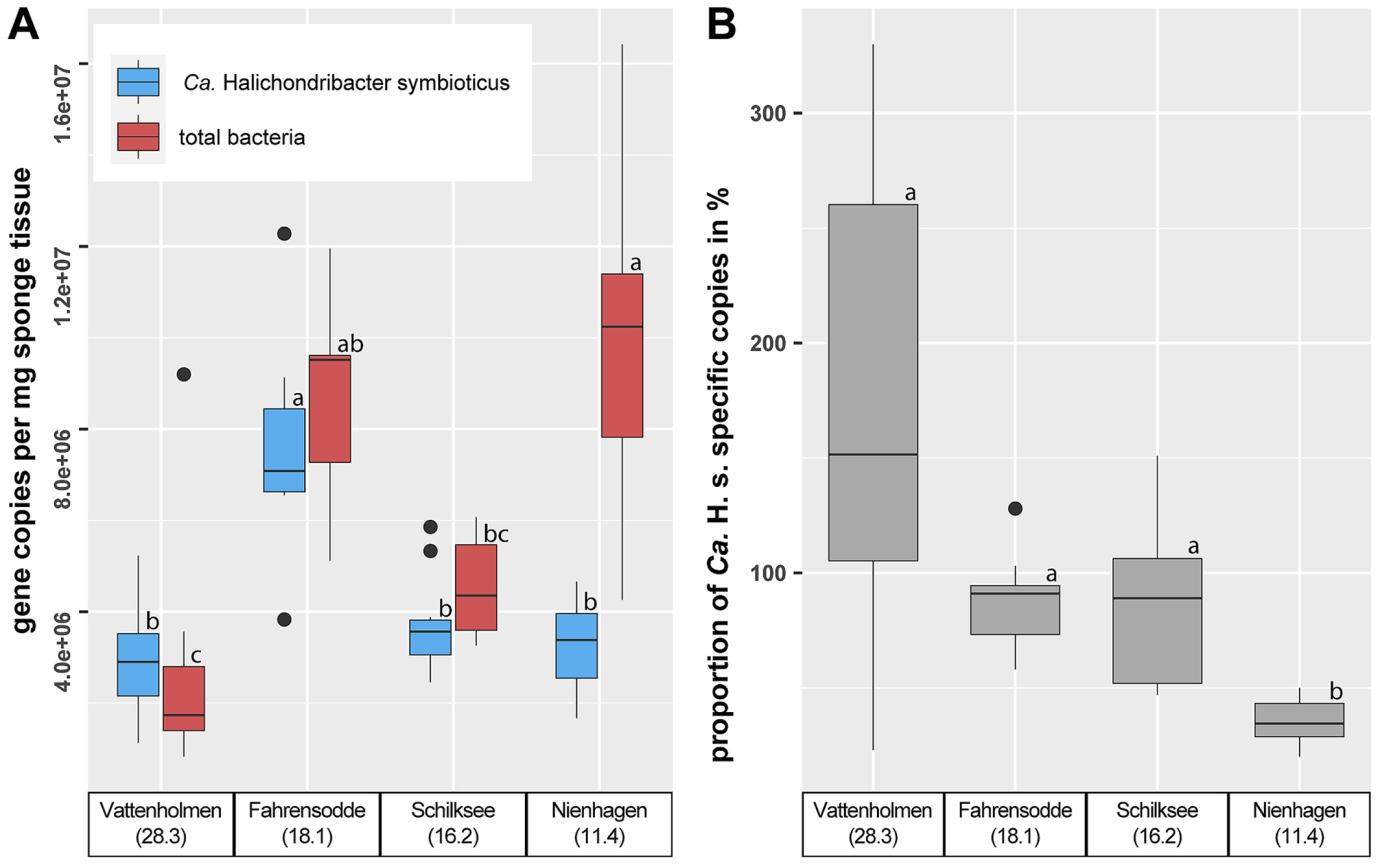
**(A)** Absolute quantification of *Ca. Halichondribacter symbioticus* and the total bacterial community in sponge microbiomes by copy numbers of 16S rRNA gene determined by ddPCR. **(B)** Ratio of *Ca.* H. s. to total bacterial community in %. Different lowercase letters indicate significant differences.

16S rRNA gene copy numbers of *Ca.* H.s., as well as those of the total bacterial community, determined by ddPCR, showed significant differences by location (Kruskal–Wallis: *χ*^2^ = 20.807, df = 3, *p*-value < 0.001 and *χ*^2^ = 26.756, df = 3, *p*-value < 0.001, respectively) (Figure 5A). Sponge microbiomes from Fahrensodde exhibited nearly twice as many 16S rRNA gene copy numbers for *Ca.* H.s. compared to the other three locations, which showed no significant differences (Figure 5A; Supplementary Table S6). These trends were also seen for total bacterial 16S rRNA gene copies, which were twice as high within sponges from Fahrensodde compared to Vattenholmen and Schilksee. Only Nienhagen stood out and showed a threefold increase in absolute abundance of the total bacterial community in relation to *Ca.* H.s. (Figure 5A). The ratio between copy numbers for *Ca.* H.s. and copy numbers for the total bacterial community also displayed a significant difference by location (Kruskal–Wallis: *χ*^2^ = 21.054, df = 3, *p*-value < 0.001) (Figure 5B). Sponge microbiomes from Vattenholmen showed the highest ratios, even exceeding 100% according to the recorded data, but also the highest variability between samples (23–330%). Nevertheless, Vattenholmen, Fahrensodde, and Schilksee did not show statistically significant differences in their ratios. Only the sponge microbiomes originating from Nienhagen exhibit a statistically significantly higher bacterial load in relation to *Ca.* H.s. (Figure 5B; Supplementary Table S6).

### Other potential host-specific bacterial taxa

For analysing the distribution and abundance of potential other sponge-specific taxa besides *Ca.* H.s., we focused on the 50 most abundant ASVs of the sponge microbiomes, using the estimated ASV copy numbers (Supplementary Table S7).

**Figure 6:**
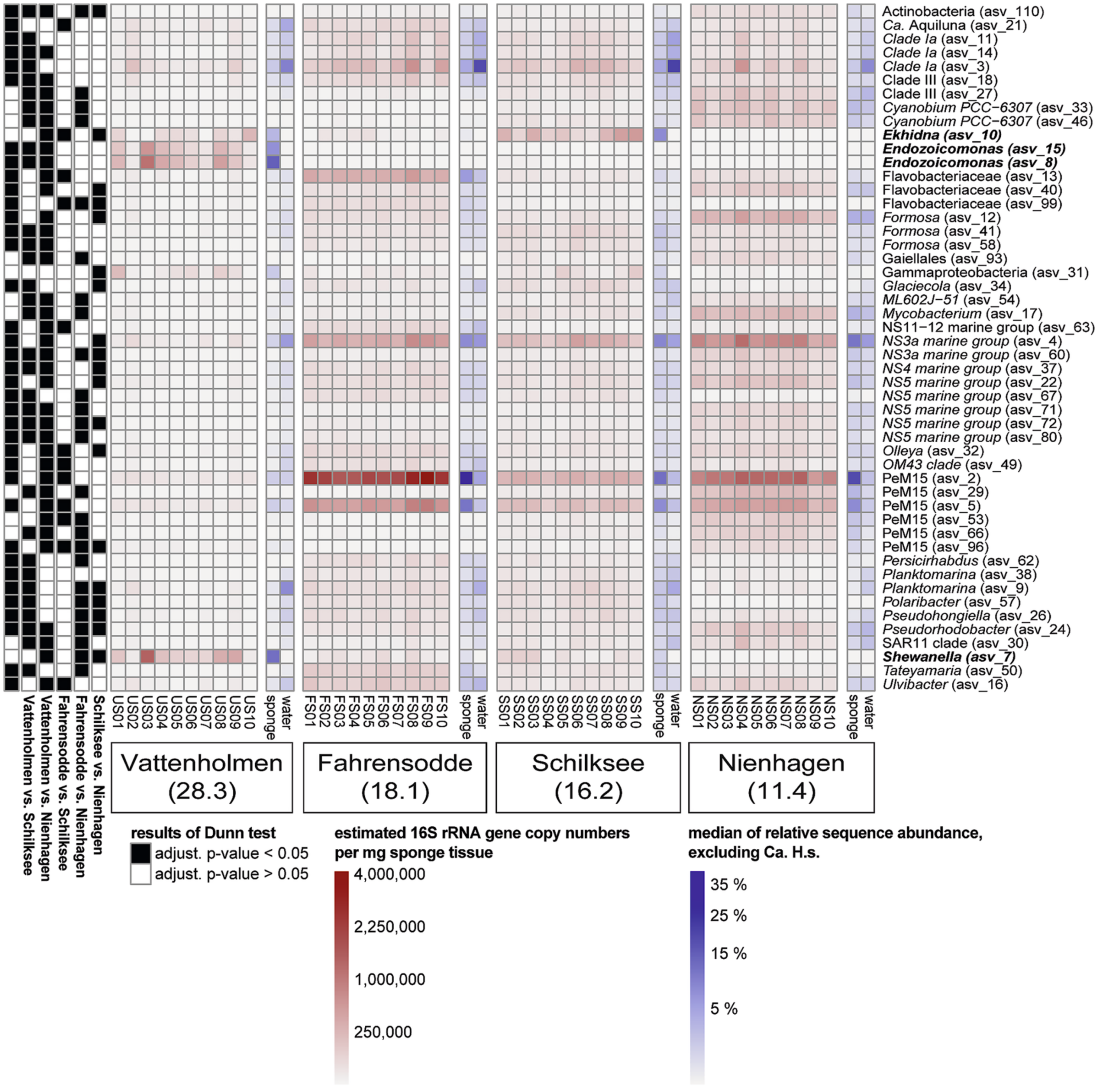
Heatmap of calculated estimates of 16S rRNA gene copy numbers of the 50 most abundant ASVs along with the median of their relative abundance in sponges and seawater, excluding *Ca.* H. s. Results of Kruskal–Wallis tests and complete taxonomic information of the ASVs are shown in Supplementary Table S8. Results of *post-hoc* test (Dunn) are shown as a binary heatmap on the left (black: significant; white: non-significant).

All 50 most abundant ASVs of the sponge microbiomes were differently abundant between locations (Supplementary Table S8), and together represented between 39.8 and 91.4% of the remaining sponge microbiomes, when *Ca.* H.s. was excluded (Supplementary Table S9). Among the top 50 ASVs, several were present in higher abundance both within the sponge and the water, such as SAR11 clade 1a, the *Flavobacteriaceae* NS3 marine group, and ASVs of the *Actinobacteria* PeM15 group (with 6 ASVs). However, most sponges were host to further potential sponge-specific taxa that occurred barely or not at all in the surrounding seawater. This was especially obvious for ASVs from the three bacterial genera *Endozoicomonas* (ASVs 8 and 15), *Shewanella* (ASV 7), and *Ekhidna* (ASV 10), which had high copy numbers in sponges from some of the locations and were mostly rare or absent in seawater. At Vattenholmen, where many ASVs had significantly lower copy numbers compared to the other three locations (Figure 6), ASVs of all three genera were enriched with maximal median estimated copy numbers of 2.73 × 10^5^ (*Endozoicomonas*), 1.29 × 10^5^ (*Shewanella*), and 3.84 × 10^4^ (*Ekhidna*) per sponge individual. Remarkably, 2–3 sponge individuals deviated from the other sponges and had a much lower abundance of both *Endozoicomonas* and *Shewanella* ASVs (Figure 6). The *Ekhidna* ASV achieved high estimated copy numbers (median 1.95 × 10^5^) also in Schilksee sponges, whereas the *Shewanella* ASV was enriched in sponges from Fahrensodde (median 1.54 × 10^4^) and Schilksee (median 2.32 × 10^4^). In most sponges from the location Nienhagen, none of these three presumably sponge-associated ASVs were found. Here, instead, typical seawater bacterial taxa that were present also in the surrounding seawater, dominated, such as several ASVs of SAR11 clades, the NS3a and PeM15 groups, and two *Cyanobium* ASVs.

## Discussion

In this study, we aimed to investigate the influence of the natural salinity gradient in the south-western Baltic Sea on the microbiome of the dominant sponge *H. panicea*. The four locations of our sampling covered a salinity range, from nearly fully marine conditions in the Kattegat (28.3 Vattenholmen) to brackish water conditions at the westernmost location in the Bay of Mecklenburg (11.3, Nienhagen), presumably close to the distribution limit of *H. panicea* in the Baltic Sea (Zettler et al., 2018). The molecular marker used for confirming sponge identification revealed minimal intraspecific diversity among the sampled individuals, with only two haplotypes differing in one base pair. This study is the first investigation of sponge microbiomes in the Baltic Sea on a larger geographical scale. Previously, only *H. panicea* microbiome samples from the Kiel Bight and Schilksee had been examined (Althoff et al., 1998; Schmittmann et al., 2022). To our knowledge, it is also among the first studies examining a sponge microbiome across a larger natural salinity gradient. In an experimental study, salinity fluctuations within 36 to 25 PSU revealed no major impact on the microbiomes of six marine sponge species (Glasl et al., 2018).

The sponge microbiomes of all analysed sponge specimens were dominated by the known symbiont *Ca.* H.s., comprising between 27 and 91% of the prokaryotic communities. This is well in line with previous studies on the microbiome of *H. panicea* in the North Sea (Wichels et al., 2006; Naim et al., 2014), the North Atlantic (Knobloch et al., 2019), and Kiel Bight in the Baltic Sea (Althoff et al., 1998; Schmittmann et al., 2022). Even though studies prior to Knobloch et al. (2019) referred to the dominant taxon simply as *Alphaproteobacterium*, a relative of *Rhodobacter* or member of the *Roseobacter* group, a high sequence similarity provides evidence that all mentioned studies refer to the same bacterial symbiont (Knobloch et al., 2019). Using similar methodology, *Ca.* H. s. constituted on average 80.5% of the microbiome in the Eastern Scheldt (Netherlands, North Sea) (Naim et al., 2014), 72% in Icelandic waters (Knobloch et al., 2019), and 36–54% in Kiel Fjord (Schmittmann et al., 2022). This suggests that higher salinities are associated with higher proportions of *Ca.* H.s. in the *H. panicea* microbiomes. Similarly, in this study, we found the mean relative proportions to decrease from 65.9% in the highest salinity station Vattenholmen to 55.7–59% in Fahrensodde and Schilksee, down to 34.5% in the most eastern station Nienhagen (Figure 2). This decreasing trend was confirmed by the absolute quantification with ddPCR, with Nienhagen having a significantly lower proportion of *Ca.* H. s. (Figure 5). However, interpretation of the 16S rRNA gene data along with the ddPCR data revealed that the absolute abundance of *Ca.* H.s. was in the same range in Vattenholmen, Schilksee, and Nienhagen, while the decrease in proportion was a result of an increase in total bacterial abundance (Figure 5). At Fahrensodde, both the absolute abundance of *Ca.* H.s. and total bacterial community doubled compared to Vattenholmen and Schilksee, while the ratio of *Ca.* H.s. to the total bacterial community remained similar to Schilksee. The cause of this higher bacterial abundance at Fahrensodde might be due to hitherto unknown environmental factors. The larger variability of the results from the Vattenholmen samples compared to the other stations, as well as differences in composition, might be due to different environmental factors, although we cannot rule out that differences in sampling (year, fixative) affected the results. However, we would argue that previous studies with comparable results also used RNAlater as a fixative, as well as sponges collected from different seasons and years (Schmittmann et al., 2022), and suspect effects to be minor compared to differences in location and their associated environmental conditions.

The relative proportions from the 16S rRNA gene amplicon sequencing data and the absolute quantification of copy numbers using ddPCR provide different but complementary insights. However, we are aware that both methods are biased due to differences in DNA extraction and PCR amplification efficiencies between different taxa, and therefore, our calculation of 16S rRNA gene copy numbers can only be an estimate. Probably due to the higher efficiency of the specific *Ca.* H.s. primers compared to total bacterial primers, the calculated proportion of *Ca* H.s. exceeded 100% in some cases (Figure 5, see also Schmittmann and Pita, 2022). Conversely, it may be that the 16S rRNA gene amplicon sequencing data underestimate the actual contribution of *Ca.* H.s. This would be in line with the study by Knobloch et al. (2019), who used fluorescence *in situ* hybridisation (FISH) with specific probes as another quantification of *Ca.* H.s., and showed that the sponge mesohyl had a much higher proportion of *Ca.* H.s. than derived from the 16S rRNA gene compositional data.

One goal of this study was to examine whether, besides *Ca.* H.s., other sponge-specific taxa occur that are present along the entire salinity gradient. Although ASVs of several other bacterial genera (*Endozoicomonas, Shewanella, Ekhidna*) were enriched in some of the locations relative to the surrounding water (Figure 6), none of those were found at all locations and therefore could probably not constitute part of the general sponge-specific community of *H. panicea*. However, their absence in the surrounding seawater identifies the sponge tissue as a favored habitat over the seawater for these taxa at the respective locations. Similar to our study, *H. panicea* from different localities of Icelandic waters harbored additional, enriched bacterial taxa, which did not occur at all sites and sponge individuals (Knobloch et al., 2019). Also, in the experimental study by Schmittmann et al. (2022), several bacterial taxa, different than the ones reported here, were permanently associated with *H. panicea* in addition to *Ca.* H.s. One reason might be that different relevant environmental factors, such as water depth, temperature, and nutrient concentrations, can affect seasonal and spatial changes of low-abundant microbial taxa in sponge microbiomes (Gan et al., 2024).

Information from the literature suggests that these enriched bacterial taxa could also potentially be functional symbionts in *H. panicea*. The genus *Endozoicomonas* is widely known for diverse symbioses with marine invertebrates, including sponges (Gardères et al., 2015; Neave et al., 2016). Some representatives of the genus *Shewanella* are known as common sponge symbionts, with adaptations to a host-dependent lifestyle (Alex and Antunes, 2019) and were also found in the microbiome of *H. panicea* (Schmittmann et al., 2022; Wichels et al., 2006). The genus *Ekhidna* comprises both free-living as well as host-dependent species (Alain et al., 2010). In the past, this genus has been detected in the microbiome of the sponge *Lamellodysidea herbacea* and is possibly responsible for the biosynthesis of phenolic lipids (Podell et al., 2020). Interestingly, there was a large variability in the contribution of these taxa to the sponge microbiomes, not only between locations but also within a location, including some sponge individuals without elevated abundance of those ASVs. Shifts or disturbances in the physiology of the sponges and resulting impacts on the microbiome might be an underlying reason here (Botté et al., 2023). It remains to be investigated whether these other taxa contribute to the functional redundancy of sponges, in which important metabolic functions are maintained across large spatial gradients by a variable host–microbe network (Busch et al., 2022a; Lurgi et al., 2019).

Along the decreasing salinity gradient from the Kattegat into the Baltic Sea, we observed several indications of increasing disturbance of the sponge microbiome, especially at the most eastern station Nienhagen: besides the already discussed increase in non *Ca* H.s. bacterial load and a decrease of *Ca.* H.s. proportion (Figure 5), an increasing similarity between water and sponge microbiomes (Figures 2, 3), as well as increasing alpha-diversity (Figure 4), was found. These changes in the microbiome coincided with a decrease in body size of *H. panicea* along the salinity gradient, especially visible at the Nienhagen site (Supplementary Figure S4). Salinity stress is often accompanied by a decline in body size of marine metazoans (Westerbom et al., 2002). We assume that these alterations in sponge morphology and microbiome are indications of impaired growth conditions and increased physiological stress of the host due to low salinity. These are potential early signs of a dysbiosis at this location, where *H. panicea* is at the limit of its distribution in the Western Baltic Sea (Zettler et al., 2018), probably set by the salinity level.

Investigations on the microbiomes of different sponge taxa have identified several environmental factors that potentially drive changes in microbiomes. Among those are water depth (Morrow et al., 2016; Steffen et al., 2022), salinity fluctuations (Glasl et al., 2018), anoxia (Schuster et al., 2021), or a combination of factors such as temperature, salinity, and oxygen (Busch et al., 2022a). However, we are not aware of a study that examined the sponge holobiont at the limit of its distribution along an environmental gradient. Studies that investigated the impact of anthropogenic climate change, with environmental alterations such as warming, acidification, and deoxygenation, revealed information on stress responses of sponge microbiomes. Although there are large differences among the examined sponge taxa (e.g., Posadas et al., 2022), some patterns emerged, which might also be valid when looking at sponge microbiomes at the edge of environmental gradients, as in this study. For example, simulated heat waves resulted in increased alpha diversity in sponge microbiomes (e.g., Luter et al., 2012), a shift toward a community more similar to the surrounding seawater (De Castro-Fernández et al., 2023) or sediment community (Williams et al., 2024), and a loss of different, microbiome-mediated functions (Botté et al., 2023). Despite these changes, the dominant taxa of the “core community” were often maintained in the host. This was also obvious in an experimental study with *H. panicea*, where antibiotic treatment induced dysbiosis and promoted a strong increase of opportunistic bacteria while *Ca.* H. s. was maintained at stable abundances (Schmittmann et al., 2022). Overall, these observations fit our findings where the absolute abundance of the main symbiont *Ca.* H.s. at the presumably salinity-stressed site Nienhagen was not lowered, but a higher proportion of other bacterial taxa from the environment were present.

All marine organisms in the Baltic Sea are facing physiological challenges due to the declining salinity and meet their distribution limit somewhere along the 2,000 km spanning salinity gradient (Ojaveer et al., 2010; Snoeijs-Leijonmalm, 2017; Kivistik et al., 2020). Especially for osmoconforming invertebrates, this is often due to their inability to further adjust their intracellular osmolality in response to the environment (Podbielski et al., 2022). However, the effect on the associated microbiomes is presently unknown. A physiologically stressed sponge host might gradually lose its ability to maintain a stable microbiome, resulting in a dysbiotic microbiome as induced by other stressors. However, despite the increasing proportion of seawater bacteria in the sponge microbiome at the low salinity scenario, a consistent population of the main symbiont *Ca.* H.s. was maintained. It remains to be investigated whether the main functions of the microbiome are impacted at this stage, and how much the sponge host is impaired physiologically by the low salinity and changes in the microbiome. Such knowledge would also help to evaluate the impact of other ongoing stressors in the Baltic Sea, besides fluctuations in salinity, such as eutrophication and pollution, on the physiology and ecological performance of *H. panicea*.

## Data Availability

The 16S rRNA gene sequence data and ddPCR data for this study have been deposited in the European Nucleotide Archive (ENA) at EMBL-EBI under accession number PRJEB77370, using the data brokerage service GFBio (Diepenbroek et al., 2014). The complete R scripts used for the 16S rRNA gene amplicon sequence data and ddPCR data analysis can be found in the corresponding git repository (Hassenrueck and Hoy, 2023). The cytochrome c oxidase I gene sequence data for this study have been deposited in the National Center for Biotechnology Information (NCBI) Genbank under accession numbers PP728111 - PP728150. Simulated salinity data was accessed from the The Federal Maritime and Hydrographic Agency (BSH) and is publicly available via the open dataportal https://gdi.bsh.de/de/feed/Data-of-the-Operational-Models.xml.
