# Supplementary material for: Microbiome changes in the sponge Halichondria panicea along the Baltic Sea salinity gradient
Source: Front Microbiol. 2026 Jan 27;16:1723082. doi: 10.3389/fmicb.2025.1723082 (PMC12890254; doi:10.3389/fmicb.2025.1723082)
Supplement: Supplementary file 1 [file Data_Sheet_1.pdf]

## Supplementary Information – Figures and Tables

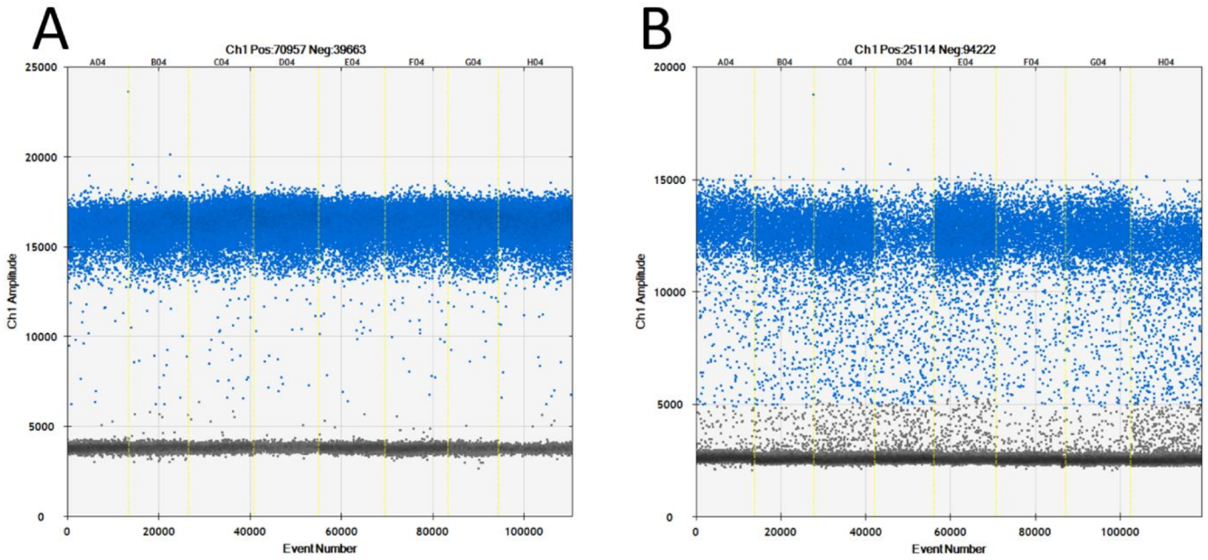


**Supplementary Figure S1:** Fluorescence intensity of partial 16S rRNA gene fragments amplified in ddPCR with DNA extracts of sponge tissue. (A): ”Hal Sym” primer set. (B): ”E1052f+E1193r” primer set. The more distinct bands with fewer noise indicate a more precise and reliable quantification with the “Hal Sym” primer set compared to the ”E1052f+E1193r” primer set.

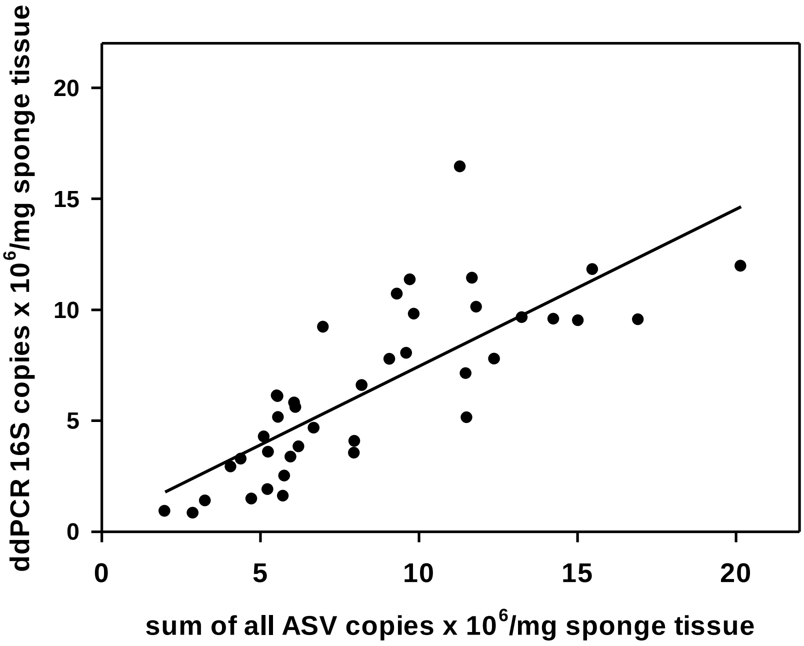


**Supplementary Figure S2:** Correlation between sum of calculated estimates of copy numbers for all ASVs (per mg sponge tissue) and ddPCR results with bacterial 16S primers (per mg sponge tissue), for each sponge sample separately (Pearson correlation coefficient: 0.785; P < 001).

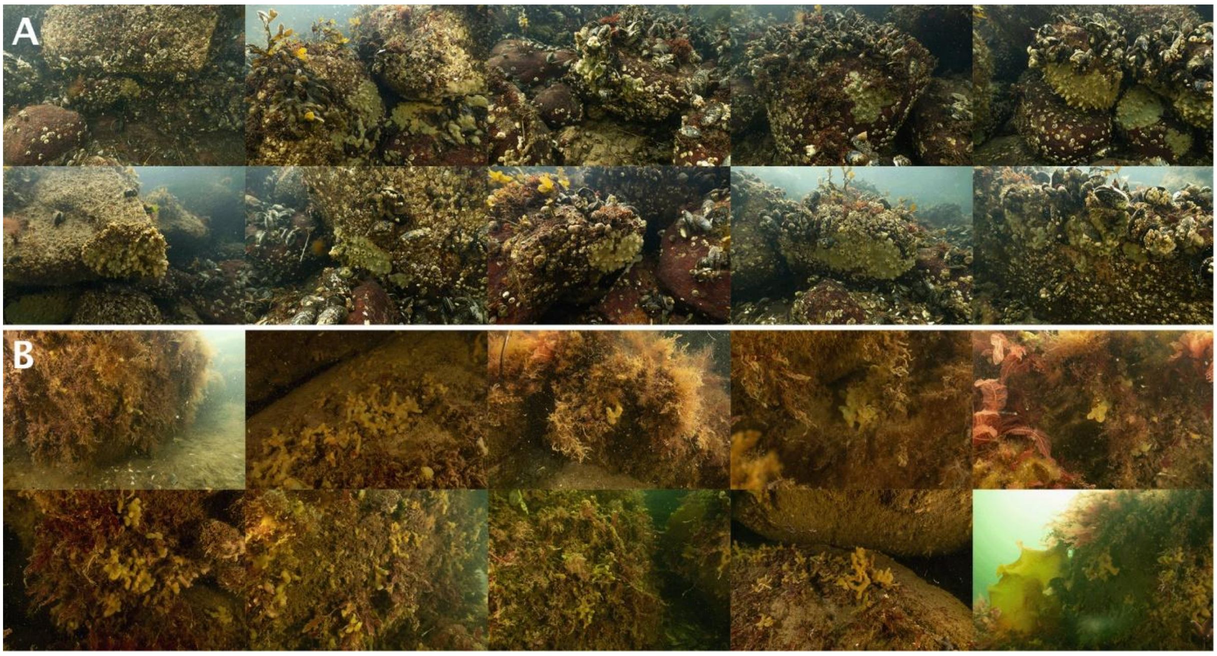


**Supplementary Figure S3:** *In situ* images of sponge individuals sampled in German waters. (A): Fahrensodde, (B): Schilksee. *In situ* images of sponges sampled at Vattenholmen and Nienhagen were not available.

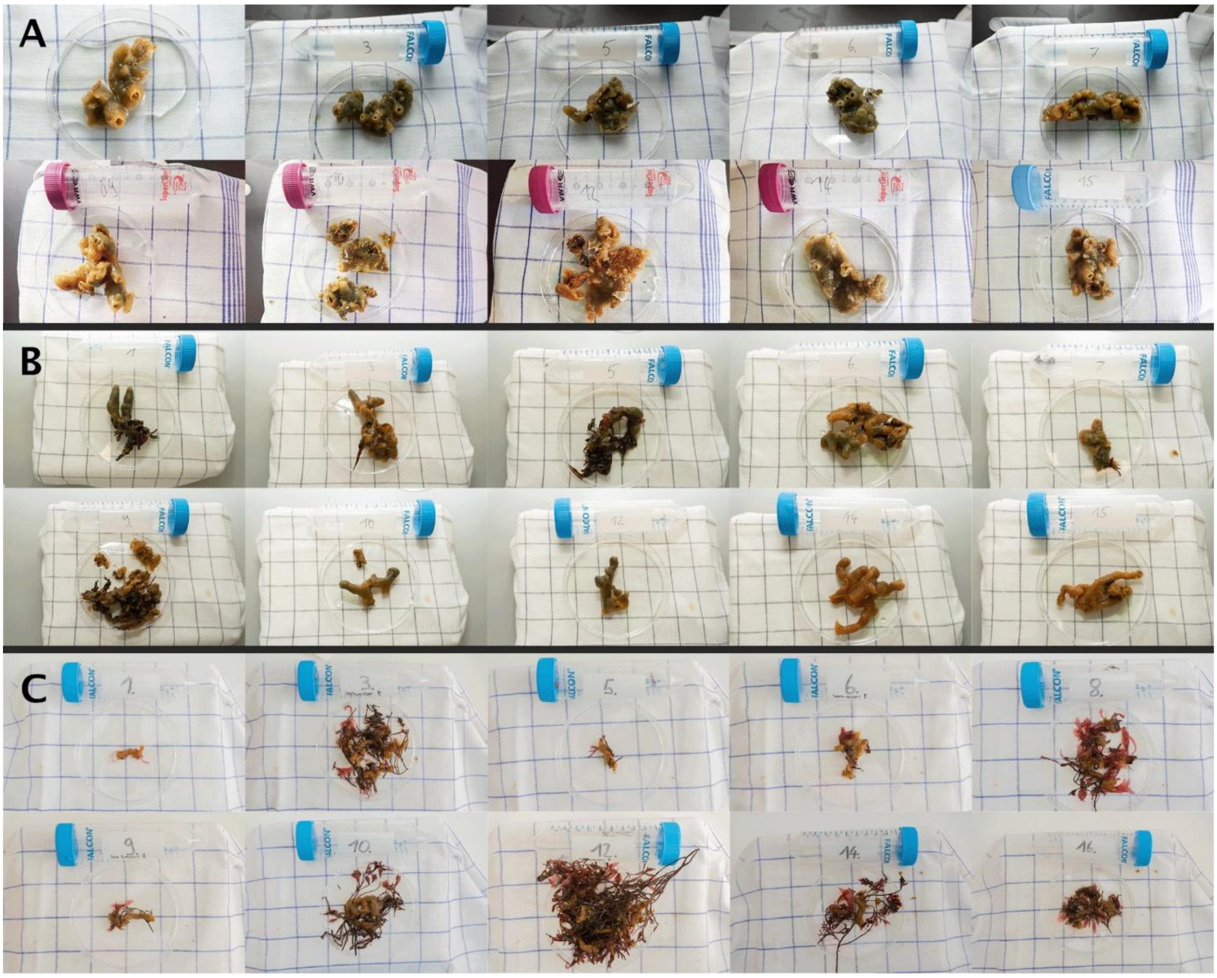

**Supplementary Figure S4:** *Ex situ* images of sponge individuals sampled in German waters. (A): Fahrensodde, (B): Schilksee, (C): Nienhagen. *Ex situ* images of sponges sampled at Vattenholmen were not available.

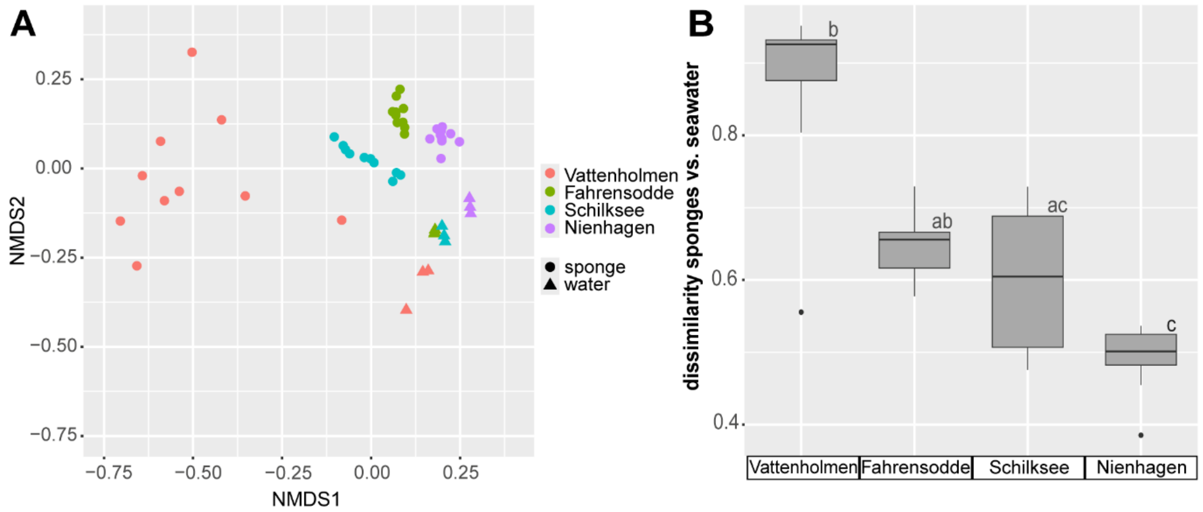


**Supplementary Figure S5:** (A): Beta diversity of sponge and seawater microbiomes based on 16S rRNA gene sequence dataset excluding *Ca.* H.s. sequences (PERMANOVA: $R^2 = 0.512$, $F = 12.589$, $p = 0.01$). Clustering was performed using nonmetric multi-dimensional scaling (NMDS) of Bray-Curtis dissimilarities. (B): Bray-Curtis dissimilarities based on 16S rRNA gene sequence data, excluding *Ca.* H.s. sequences, between individual sponge microbiomes and the centroid of corresponding seawater microbiomes per location. Results of post-hoc test (Dunn) are shown as compact letter display.

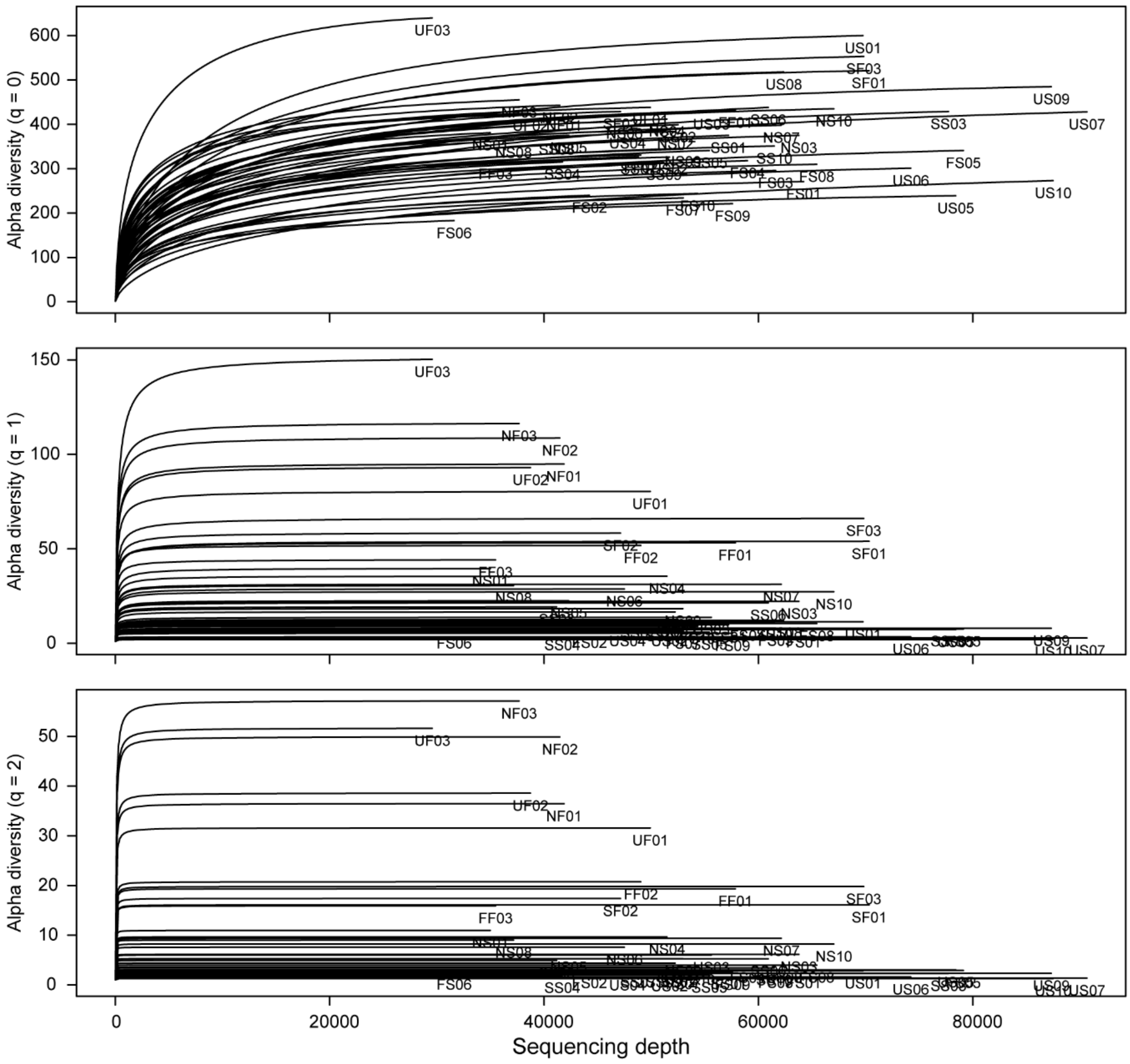


**Supplementary Figure S6:** Rarefaction curve for richness (q = 0), Shannon diversity (q = 1) and Simpson diversity (q = 2) of the 16S rRNA gene sequence dataset.

**Supplementary Table S1: Estimated 16S rRNA primer coverage, using silva TestPrime1.0 (SILVA 138.2, Nov. 2025)**

Results were derived using two different settings: a) strict: maximum number of mismatches: 1; length of 0-mismatch zone at 3´end: 5; b) relaxed: maximum number of mismatches: 3; length of 0-mismatch zone at 3´end: 3.

| | | Coverage (%) | | | |
|---|---|---|---|---|---|
| Application | Primer | Bacteria | | Archaea | |
| | | strict | relaxed | strict | relaxed |
| Amplicon sequencing | Sundberg 341F/806R | 87.3 | 92.5 | 86.3 | 93.6 |
| ddPCR | E1052f/E1193r | 91.1 | 95.4 | 0.0 | 37.0 |

Examining the Hal Sym primers in Silva TestPrime1.0 produced a match with only two sequences within the genus *Amylibacter* (accession numbers MH734529, AY948354).

The ddPCR 16S rRNA gene primers also matched these two sequences (both in strict and relaxed mode).

A Blastn search with these two sequences in NCBI revealed partial sequences of *Candidatus* Halichondribacter symbioticus (clones Hp2 and Hp1) with 98.99-100% identity. Few other partial sequences, mostly sponge clones, had also similarities > 98%.

**Supplementary Table S2:** Test report of ANOSIM of beta diversity of sponge microbiomes. P-values adjusted with the FDR method. Significant p-values (< 0.05) are highlighted in bold.

| | incl. *Ca.* Halichondribacter | | excl. *Ca.* Halichondribacter | |
|---|---|---|---|---|
| | ANOSIM R | ANOSIM Padj. | ANOSIM R | ANOSIM Padj. |
| Vattenholmen - Fahrensodde | 0.768 | **0.001** | 0.908 | **0.001** |
| Vattenholmen - Schilksee | 0.604 | **0.001** | 0.795 | **0.001** |
| Vattenholmen - Nienhagen | 0.951 | **0.001** | 0.946 | **0.001** |
| Fahrensodde - Schilksee | 0.813 | **0.001** | 0.969 | **0.001** |
| Fahrensodde - Nienhagen | 1.000 | **0.001** | 1.000 | **0.001** |
| Schilksee - Nienhagen | 0.979 | **0.001** | 0.982 | **0.001** |

**Supplementary Table S3:** Test report of Dunn-test of Bray-Curtis dissimilarity between individual sponge samples and corresponding seawater microbiome between locations. P-values adjusted with the Bonferroni method. Significant p-values (< 0.05) are highlighted in bold.

| Comparison | Z | P.adj |
|---|---|---|
| Fahrensodde - Nienhagen | 2.946 | **0.019** |
| Fahrensodde - Schilksee | 0.459 | 1.000 |
| Nienhagen - Schilksee | -2.487 | 0.077 |
| Fahrensodde - Vattenholmen | -2.334 | 0.118 |
| Nienhagen - Vattenholmen | -5.279 | **<0.001** |
| Schilksee - Vattenholmen | -2.793 | **0.031** |

**Supplementary Table S4:** Test report of Dunn-test of Bray-Curtis dissimilarity between individual sponge samples and corresponding seawater microbiome between locations, excluding *Ca*. H.s. P-values adjusted with the Bonferroni method. Significant p-values (< 0.05) are highlighted in bold.

| Comparison | Z | P.adj |
|---|---|---|
| Fahrensodde - Nienhagen | 2.869 | **0.025** |
| Fahrensodde - Schilksee | 0.937 | 1.000 |
| Nienhagen - Schilksee | -1.932 | 0.320 |
| Fahrensodde - Vattenholmen | -2.123 | 0.202 |
| Nienhagen - Vattenholmen | -4.992 | **<0.001** |
| Schilksee - Vattenholmen | -3.060 | **0.013** |

**Supplementary Table S5:** Test report of Dunn-test of alpha diversity indices. P-values adjusted with the Bonferroni method. Significant p-values (< 0.05) are highlighted in bold.

| **richness** | | |
|---|---|---|
| Comparison | Z | P.adj |
| Fahrensodde - Nienhagen | -3.749 | **0.001** |
| Fahrensodde - Schilksee | -2.850 | **0.026** |
| Nienhagen - Schilksee | 0.899 | 1.000 |
| Fahrensodde - Vattenholmen | -3.271 | **0.006** |
| Nienhagen - Vattenholmen | 0.478 | 1.000 |
| Schilksee - Vattenholmen | -0.421 | 1.000 |
| **exponential Shannon index** | | |
| Comparison | Z | P.adj |
| Fahrensodde - Nienhagen | -4.265 | **<0.001** |
| Fahrensodde - Schilksee | -1.396 | 0.976 |
| Nienhagen - Schilksee | 2.869 | **0.025** |
| Fahrensodde - Vattenholmen | -0.230 | 1.000 |
| Nienhagen - Vattenholmen | 4.036 | **<0.001** |
| Schilksee - Vattenholmen | 1.167 | 1.000 |

**Supplementary Table S6:** Test report of Dunn-tests of ddPCR data. P-values adjusted with the Bonferroni method. Significant p-values (< 0.05) are highlighted in bold.

| **Copy numbers *Ca*. Halichondribacter symbioticus** | | |
|---|---|---|
| Comparison | Z | P.adj |
| Fahrensodde – Nienhagen | 3.558 | **0.002** |
| Fahrensodde – Schilksee | 3.022 | **0.015** |
| Nienhagen – Schilksee | -0.536 | 1.000 |
| Fahrensodde – Vattenholmen | 4.208 | **<0.001** |
| Nienhagen – Vattenholmen | 0.650 | 1.000 |
| Schilksee – Vattenholmen | 1.186 | 1.000 |
| **Copy numbers total bacterial community** | | |
| Comparison | Z | P.adj |
| Fahrensodde – Nienhagen | -0.440 | 1.000 |
| Fahrensodde – Schilksee | 2.563 | 0.062 |
| Nienhagen – Schilksee | 3.003 | **0.016** |
| Fahrensodde – Vattenholmen | 3.998 | **<0.001** |
| Nienhagen – Vattenholmen | 4.438 | **<0.001** |
| Schilksee – Vattenholmen | 1.435 | 0.908 |
| **Proportion *Ca*. Halichondribacter symbioticus** | | |
| Comparison | Z | P.adj |
| Fahrensodde – Nienhagen | 3.061 | **0.013** |
| Fahrensodde – Schilksee | 0.086 | 1.000 |
| Nienhagen – Schilksee | -2.975 | **0.018** |
| Fahrensodde – Vattenholmen | -1.387 | 0.993 |
| Nienhagen – Vattenholmen | -4.448 | **<0.001** |
| Schilksee – Vattenholmen | -1.473 | 0.844 |

**Supplementary Table S7:** Median of estimated absolute copy numbers and relative sequence abundance for each of the 50 most abundant ASVs excluding Ca. H.s. (**see Excel Table S7**)

**Supplementary Table S8:** Test report of Kruskal-Wallis-tests of estimated copy numbers of top 50 ASVs by location, and taxonomic information on top 50 ASVs. P-values adjusted with the FDR method. Significant p-values (< 0.05) are highlighted in bold. (**see Excel Table S8**)

**Supplementary Table S9:** Mean percentages of the prokaryotic communities within the sponge tissues represented by the 50 most abundant ASVs, excluding Ca. Halichondribacter.

| Location | Min. | 1st Qu. | Median | Mean | 3rd Qu. | Max. |
|---|---|---|---|---|---|---|
| Vattenholmen | 39.79 | 57.21 | 59.39 | 59.40 | 62.00 | 73.19 |
| Fahrensodde | 84.31 | 87.15 | 87.79 | 87.89 | 89.39 | 91.35 |
| Schilksee | 66.40 | 74.97 | 77.78 | 76.30 | 78.58 | 82.13 |
| Nienhagen | 69.20 | 74.46 | 77.13 | 75.94 | 77.90 | 80.65 |
